# Revising the WHO Essential Medicines List for paediatric rheumatology

**DOI:** 10.1186/s12969-021-00496-3

**Published:** 2021-01-23

**Authors:** Christiaan Scott, Nicola Smith, Rebecca James, Ben Whitehead, Rochelle Green, Helen E. Foster, on behalf of the Paediatric Global MSK Task Force

**Affiliations:** 1 Paediatric Rheumatology, University of Cape Town, Cape Town, South Africa; 2 Translational and Clinical Research Institute, Newcastle University, Newcastle upon Tyne, UK; 3 Paediatric Rheumatology, Queensland Children’s Hospital, Brisbane, Queensland Australia; 4 Pharmacy, Queensland Children’s Hospital, Brisbane, Queensland Australia; 5 Population and Health Sciences Institute, Newcastle University, Newcastle upon Tyne, UK

Dear Editor,

The World Health Organisation (WHO) Essential Medicines List (EML) [1] informs countries about the minimum medicine items necessary to meet priority health needs of the population and guide national and institutional medicine lists, especially in Low Resource Income Countries. The current EML under medicines for ‘joint diseases in children’ does not reflect current best practice [2] and an important theme of work from the Paediatric Global Musculoskeletal Health Task Force (TF) [3] is to revise the listing for medicines relevant to paediatric rheumatic diseases.

Healthcare professionals working in paediatric rheumatology and who are TF members were invited to take part in an anonymous online survey WHO EML to explore which drugs they deemed to be ‘essential’ and ‘ideal’ for the clinical practice in their context. No reminders to the survey were sent. We had 97 responders, from 43 countries across all continents and mainly from low resource countries (Asia *n* = 51/97). Respondents had a range of 1–35 years of clinical practice and included consultant grade paediatric rheumatologists (*n* = 77), consultant general paediatricians with interest in rheumatology (*n* = 13), paediatric rheumatology trainees (*n* = 3), adult rheumatologists (*n* = 3) and a nurse working in paediatric rheumatology (*n* = 1). Survey data were analysed by applying descriptive statistics and free-text comments were analysed following standard procedures for qualitative analysis [4].

Most respondents (*n* = 70/97, 72%) reported that a revised EML would very likely improve access to medicines in their country, improve drug accessibility within their clinical practice, provide assistance when negotiating with healthcare agencies or insurance companies and further increase awareness about paediatric rheumatology issues. They deemed that the EML should list the drugs in Table 1; 80% respondents identified 5 agents as ‘essential’ (oral, intra-articular and intravenous corticosteroids, NSAIDS, Hydroxychloroquine and Methotrexate [oral and subcutaneous]) and a wide range of synthetic and biologic DMARDS as well as other immunosuppressive agents be included. This ‘cut off’ of 80% will form the basis of the TF application to the WHO to revise the EML with the submission planned for late 2020. It is our hope that raising awareness and improving access to appropriate therapy will lead to better outcomes for children with rheumatic diseases globally and allow for a targeted treatment approach [5].

**Table 1:** Suggested medicines to be included in the WHO EML

Drug	Should Include (Ideal) (% refers to respondents)	Inclusion ‘Essential’
Oral prednisolone	100%	92%
Oral NSAIDs	99%	93%
Hydroxychloroquine	98%	88%
Intravenous Methylprednisolone	98%	83%
Methotrexate oral	96%	81%
Mycophenolate Mofetil	95%	77%
Azathioprine	94%	71%
Methotrexate subcutaneous	91%	80%
Intravenous cyclophosphamide	91%	77%
Adalimumab	91%	71%
Anakinra	90%	60%
Etanercept	87%	70%
Intra-articular corticosteroid Triamcinolone Hexacetonide	86%	64%
Intravenous Tocilizumab	86%	63%
Oral prednisolone (soluble)	86%	55%
Ciclosporin	85%	52%
Sulphasalazine	84%	51%
Subcutaneous Tocilizumab	81%	46%
Infliximab	80%	52%
Intravenous bisphosphonate (e.g. pamidronate)	76%	37%
Intra-articular corticosteroid Triamcinolone Acetonide	72%	28%
Intra-articular corticosteroid Methylprednisolone	45%	25%
Oral cyclophosphamide	41%	16%
Inhaled analgesia (nitrous oxide)	36%	15%
Thalidomide	34%	8%
**Total Respondents: 97**		

## Data Availability

All data generated or analysed during this study are included in this published article (and it's supplementary information files).

## Abbreviations

EML: Essential Medicines List
TF: Paediatric Global Musculoskeletal Task Force
WHO: World Health Organisation

## References

[1] The WHO Essential Medicines List 2019 [Available from: https://www.who.int/medicines/publications/essentialmedicines/en/.

[2] Foster HE, Scott C (2020). Update the WHO EML to improve global paediatric rheumatology. Nat Rev Rheumatol.

[3] Foster HE, Scott C, Tiderius CJ, Dobbs MB (2020). The paediatric global musculoskeletal task force-‘towards better MSK health for all’. Pediatr Rheumatol.

[4] Rapley T, Silverman D (2011). Some pragmatics of data analysis. Qualitative research:issues of theory, method and practice.

[5] Ravelli A, Consolaro A, Horneff G, Laxer RM, Lovell DJ, Wulffraat NM (2018). Treating juvenile idiopathic arthritis to target: recommendations of an international task force. Ann Rheum Dis.

